# Comparison of Symptoms and Disease Progression in a Mother and Son with Gorlin–Goltz Syndrome: A Case Report

**DOI:** 10.3390/jcm14145151

**Published:** 2025-07-20

**Authors:** Agnieszka Adamska, Dominik Woźniak, Piotr Regulski, Paweł Zawadzki

**Affiliations:** 1 Department of Oral Surgery, Medical University of Warsaw, 02-091 Warszawa, Poland; 2 Department of Cranio-Maxillofacial Surgery, Jagiellonian University Medical College, 31-008 Krakow, Poland; dwozniak@su.krakow.pl; 3 Department of Dental and Maxillofacial Radiology, Medical University of Warsaw, 02-091 Warszawa, Poland; piotr.regulski@wum.edu.pl; 4 Department of Cranio-Maxillofacial Surgery, Oral Surgery and Implantology, Medical University of Warsaw, 02-091 Warszawa, Poland; pawel.zawadzki@wum.edu.pl

**Keywords:** Gorlin–Goltz syndrome, basal cell nevus syndrome, nevoid basal cell carcinoma syndrome, *PTCH1* mutation, genetic testing, odontogenic keratocysts, basal cell carcinoma

## Abstract

**Background**: Gorlin–Goltz syndrome (GGS), also known as basal cell nevus syndrome or nevoid basal cell carcinoma syndrome, is a rare genetic disorder caused by mutations in the *PTCH1*, *PTCH2*, or *SUFU* genes, leading to an increased risk of neoplasms. Craniofacial anomalies are among the most common features of GGS. This paper aimed to highlight the similarities and differences in clinical presentation across different ages and to emphasize the importance of including all family members in the diagnostic process. The diagnosis can often be initiated by a dentist through routine radiographic imaging. **Case Presentation**: We present a 17-year longitudinal follow-up of a male patient with recurrent multiple odontogenic keratocysts and other manifestations consistent with GGS. Nearly 20 years later, the patient’s mother presented with similar clinical features suggestive of GGS. Diagnostic imaging, including contrast-enhanced computed tomography (CT), cone-beam CT, magnetic resonance imaging, and orthopantomography, was performed, and the diagnosis was confirmed through genetic testing. Interdisciplinary management included age-appropriate surgical and dermatological treatments tailored to lesion severity. **Conclusions**: Given the frequent involvement of the stomatognathic system in GGS, dentists play a critical role in early detection and referral. Comprehensive family-based screening is essential for timely diagnosis, improved monitoring, and effective management of this multisystem disorder.

## 1. Introduction

Gorlin–Goltz syndrome (GGS) was first reported in 1894 by Jarisch and White and later described in detail by Gorlin and Goltz in 1960 [1]. Since then, several alternative names for the syndrome have appeared in the literature. Initially, the condition was referred to as “nevoid basal cell epithelioma” [1]. Three years later, in 1963, Gorlin and Yunis proposed the term “nevoid basal cell carcinoma syndrome” (NBCCS), which remains widely used in the current literature [2,3]. In the same year, Herzberg and Wiskemann introduced the alternative name “basal cell nevus syndrome” (BCNS), which continues to be used interchangeably with the other terms [4].

GGS is a congenital, autosomal dominant disorder with complete penetrance and highly variable expressivity [5]. It results from a mutation of the *patched* (*PTCH1*) tumor suppressor gene [6] located on the long arm of chromosome 9 [7]. The syndrome shows no sexual predilection. Incidence rates vary depending on the population, ranging from 1 in 56,000 [7] to as rare as 1 in 13,939,393 [8]. Due to its low incidence, long-term follow-up studies are uncommon. In 2016, de Santana Santos [9] published a 10-year follow-up of a familial case series, which, at the time, represented the longest documented observation of GGS in the English-language literature. We now report a 22-year follow-up, which—to our knowledge—is the longest documented history of GGS to date.

Patients with GGS present with abnormalities affecting the skin, bones, eyes, and central nervous system. Diagnostic criteria are divided into major and minor categories. Major criteria include basal cell carcinoma (BCC) of the skin, odontogenic keratocysts (OKCs), bilamellar calcification of the falx cerebri, and rib anomalies [10]. Minor criteria encompass macrocephaly, congenital malformations, skeletal abnormalities, and certain tumors such as medulloblastoma and ovarian fibroma. The diagnosis is confirmed by the presence of either two major criteria or one major and two minor criteria [11]. A significant proportion of GGS manifestations involve the stomatognathic system. These include OKCs, dental ectopy or heterotopy, tooth impaction or agenesis, malocclusion, cleft lip or palate, mandibular prognathism, high-arched palate, and hyperplasia of the mandibular coronoid process [12].

The aim of this study was to analyze the long-term clinical progression of GGS within a single family, with a focus on differences in disease manifestation based on age at diagnosis and symptom onset. The described cases are particularly unique due to the extended observation period of over 20 years, allowing for a detailed analysis of symptom evolution and treatment effectiveness. We compared metadata regarding symptom development, diagnostic testing, and treatment processes of two family members. As genetic testing has become more accessible, the literature has reported an increasing number of familial GGS cases [13,14,15,16,17]. At the time of our first patient’s diagnosis, however, such testing was far less available and considerably more costly. Today, it is considered essential for accurate diagnosis. This case highlights how diagnostic strategies have evolved over time, particularly in the identification of GGS among family members.

## 2. Detailed Case Description

### 2.1. Case 1—Son

A 17-year-old male of European descent was referred in 2003 to the Department of Cranio-Maxillofacial Surgery, Medical University of Warsaw, Poland, with clinical suspicion of a dentigerous cyst associated with the impacted mandibular right third molar (tooth 48). The referral was initiated by his general dental practitioner following the onset of clinical symptoms, which included swelling of the right buccal and low-grade pyrexia. The patient denied experiencing any pain. His medical history was unremarkable, with no reported systemic diseases, allergies, or regular medication use. During the clinical interview, he also denied a family history of similar symptoms.

Extraoral examination revealed trismus, right-sided buccal swelling, and non-mobile right submandibular lymphadenopathy. Intraoral examination demonstrated a painless erythematous swelling of the right buccal mucosa with elevation of the mucous membrane in the lower vestibule on the same side, consistent with a presentation of an OKC associated with the impacted mandibular third molar.

**Figure 1:**
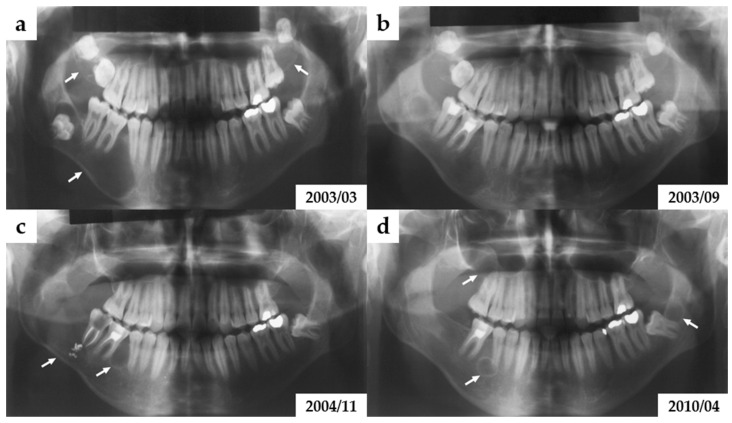
Subsequent panoramic radiographs of the son (Case 1), with OKCs indicated by arrows. (**a**) Initial orthopantomogram (March 2003) shows three radiolucent, well-defined, multilocular lesions (white arrows): one located in the right mandibular body displacing adjacent teeth and bilateral cystic lesions in the maxillary tuberosity regions, all associated with impacted third molars. (**b**) Follow-up radiograph (September 2003) after surgical removal of the mandibular lesions. Cystic lesions in the maxillae persist, (**c**) Radiograph from November 2004 reveals recurrence of mandibular lesions in the same location previously operated (marked with arrows), indicating high recurrence potential of OKCs. (**d**) In the 2010 image, new osteolytic lesions are visible in the left mandibular ramus and adjacent to the root of tooth 45 (arrows), again without evidence of root resorption. This image illustrates ongoing disease progression and recurrence despite multiple surgical interventions.

Initial panoramic radiography (orthopantomogram, OPG) and cone-beam computed tomography (CBCT) revealed three distinct osteolytic lesions: one in each maxillary tuberosity and a larger lesion in the right mandibular body. Each lesion was associated with an impacted tooth. No signs of external root resorption were present, although the mandibular lesion had displaced teeth 45 and 46 (Figure 1a).

In April 2003, biopsy and drainage of the right mandibular lesion were performed. Histopathological examination revealed keratinizing squamous epithelium with masses of keratin, chronic inflammatory granulation tissue, purulent areas, and bacterial colonies including Actinobacteria. Subsequent surgical removal of two right-sided mandibular cystic lesions and the dental follicle of tooth 48 in May 2003 confirmed the diagnosis of OKCs with associated chronic inflammation. In September 2003, additional cystic lesions were removed from the maxillae bilaterally, as well as the dental follicles of teeth 17, 18, 27, and 28.

An image from postoperative follow-up of the right mandibular body is shown in Figure 1b. Cystic lesions in the right and left maxilla, as well as the dental follicles of teeth 17, 18, 27, and 28, were removed.

Radiological follow-up in November 2004 revealed recurrent two osteolytic lesions in the mandibular body (Figure 1c). There were surgically excised in January 2005 and confirmed histologically as recurrent OKCs. Between 2010 and 2017, further recurrences were documented in both mandibles, with additional cystic lesions detected on imaging and treated surgically.

**Figure 2:**
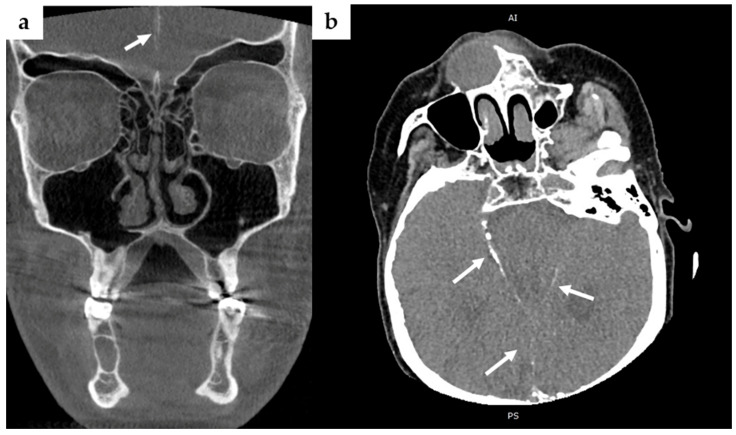
Imaging evidence of bilamellar calcification of the falx cerebri (arrows), a radiological hallmark of Gorlin–Goltz syndrome. (**a**) Coronal cone-beam CT (CBCT) scan in the son (Case 1) showing linear midline calcification along the falx cerebri; (**b**) Axial contrast-enhanced CT scan in the mother (Case 2) demonstrating extensive falx cerebri calcifications, clearly visible as linear hyperdense structures within the interhemispheric fissure.

In March 2017, CBCT imaging revealed calcifications of the falx cerebri and tentorium cerebelli—radiological hallmarks of GGS (Figure 2a).

**Figure 3:**
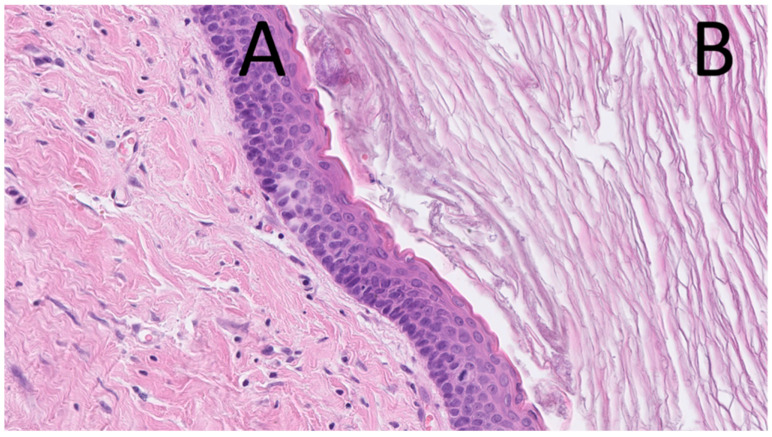
Microphotograph of the cystic lesion excised in April 2017. Hematoxylin and eosin stain. The image shows a parakeratinized stratified squamous epithelial lining (**A**) and laminated keratin masses within the cystic lumen (**B**), consistent with an odontogenic keratocyst.

Histopathological examination of lesions removed during this period consistently revealed OKCs, with both parakeratotic and orthokeratotic epithelial linings observed (Figure 3). None of the lesions showed malignant transformation or atypia.

The following clinical and radiographic features raised suspicion for a syndromic diagnosis, specifically GGS:

- Early onset and recurrent nature of OKCs;
- Multiple cystic lesions involving both the maxilla and mandible;
- Lack of external root resorption despite extensive lesions;
- Radiologically confirmed intracranial calcifications (falx cerebri and tentorium cerebelli);
- Subsequent development of cutaneous lesions consistent with basal cell carcinomas (BCCs).

Dermatological evaluations in 2016 and 2018 were unremarkable. However, in March 2022, the patient presented with nodular cutaneous lesions on the chin and cheek, characterized by rolled borders and superficial ulceration. Excisional biopsy confirmed the diagnosis of BCC.

In 2018, next-generation sequencing (NGS) was performed and identified a pathogenic heterozygous variant in the *PTCH1* gene consistent with autosomal dominant inheritance patterns. Details of the genetic findings are presented in Table 1. Mutations in PTCH1 are known to cause GGS. Based on clinical, radiological, histological, and genetic evidence, a definitive diagnosis of GGS (nevoid basal cell carcinoma syndrome) was established.

Between January 2020 and August 2022, the patient underwent four additional surgical procedures to remove recurrent or new cystic lesions in the mandible and maxilla. Histopathological analysis confirmed OKCs in all cases.

**Figure 4:**
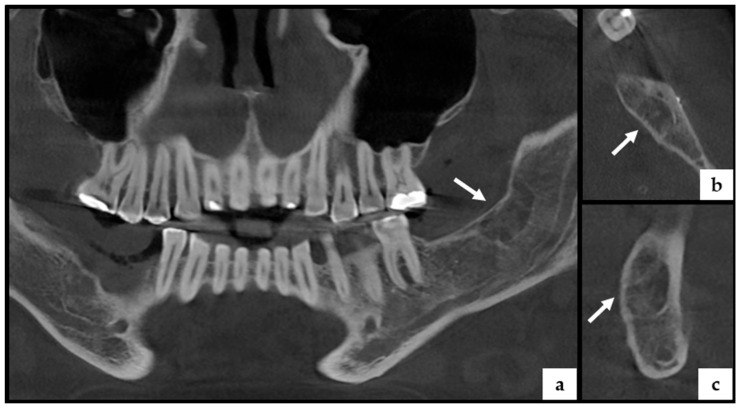
CBCT scan of the mandible (2023) in Case 1 showing no evidence of new osteolytic lesions. (**a**) Coronal/horizontal reconstruction reveals a previously affected area in the left mandibular body (arrow), now demonstrating sparse trabecular bone with partial radiodensity, consistent with bone remodeling and healing after cyst enucleation. (**b**) Sagittal view confirms a well-defined area of low bone density surrounded by sclerotic borders (arrow), indicative of previous OKC location. (**c**) Cross-sectional view through the same region shows radiolucent characteristics with peripheral bone thickening (arrow), suggesting ongoing remodeling rather than active pathology. These findings reflect post-surgical changes and stable healing, with no signs of recurrence or new cystic lesions.

Since 2023, the patient has been under routine six-month surveillance. The most recent CBCT imaging in 2025 revealed osteolytic changes in the left mandibular body with evidence of bone remodeling but no new cyst formation (Figure 4). Dermatological evaluations remain negative for new BCCs.

### 2.2. Case 2—Mother

In 2021, a 68-year-old woman of European descent was referred to the Department of Oral Surgery, Medical University of Warsaw, Poland, for the evaluation and exclusion of potential oral infection foci prior to elective ophthalmic surgery. The planned procedure involved excision of a lesion on the lower right eyelid, which was clinically suspected to be a BCC. During the preoperative evaluation and medical interview, the patient recalled that her son had received a similar diagnosis many years earlier, raising suspicion of a hereditary condition.

**Figure 5:**
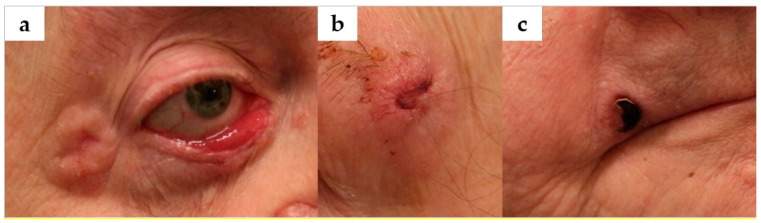
Extraoral clinical presentation in the mother (Case 2), showing multiple cutaneous lesions suspicious for basal cell carcinoma. (**a**) Nodular lesions with rolled borders on the lower right eyelid and right lateral canthus. (**b**) Ulcerated nodule in the left eyebrow region. (**c**) Dark, crusted lesion resembling a pigmented basal cell carcinoma at the right oral commissure.

Extraoral examination revealed multiple cutaneous lesions. These included nodular lesions with raised, ridge-like borders and central ulceration on the lower right eyelid and right outer canthus (Figure 5a), as well as in the left eyebrow region (Figure 5b). Some lesions demonstrated signs of healing amidst active ulceration. An additional lesion at the right oral commissure appeared as a dark scab (Figure 5c). The patient reported that these lesions would repeatedly scab over, detach, and ulcerate again—a clinical pattern typical of BCCs.

Intraoral examination showed residual roots of teeth 48 and 33. Although the root surface of tooth 33 was exposed, there was no evidence of increased mobility or acute inflammation within the oral cavity.

**Figure 6:**
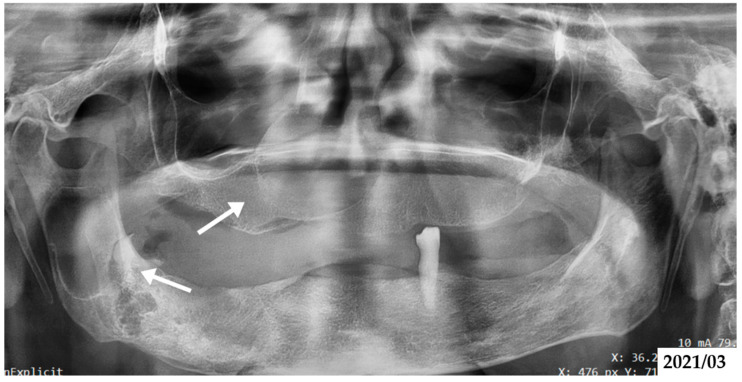
Panoramic radiograph of the mother (Case 2), taken in March 2021. Arrows indicate two well-defined radiolucent lesions in the left mandibular body and ramus, radiographically consistent with odontogenic keratocysts (OKCs).

Radiological assessment included OPG and contrast-enhanced computed tomography (CT) to evaluate the extent and nature of craniofacial lesions. These imaging modalities identified multiple osteolytic lesions in both the maxilla and mandible (Figure 6), suggestive of OKCs, as well as intracranial calcifications involving the falx cerebri and tentorium cerebelli (Figure 2b). Based on the patient’s dermatological and radiological findings, along with the relevant family history, a working diagnosis of GGS was established.

Genetic testing was performed several months after the initial clinical evaluation using a targeted NGS panel including *PTCH1*, *PTCH2*, and *SUFU*. DNA was extracted from peripheral blood, and sequencing was carried out on a high-throughput platform, with bioinformatic analysis enabling detection of all single-nucleotide variants and over 95% of small insertions and deletions. A pathogenic heterozygous variant in *PTCH1* (Table 2) was identified, confirming the diagnosis of Gorlin–Goltz syndrome. The same mutation was also detected in the patient’s son, indicating a familial case of the syndrome.

In July 2021, following genetic confirmation, the patient underwent surgical removal of osteolytic lesions from the maxilla and mandible. Concurrently, several cutaneous lesions were excised. Histopathological analysis confirmed the jaw lesions as OKCs and the skin lesions as BCCs.

This case highlights the value of comprehensive preoperative assessments in uncovering syndromic conditions and the critical role of family history and genetic testing in diagnosing Gorlin–Goltz syndrome. Although the son had been diagnosed approximately 20 years earlier, the mother’s diagnosis was delayed and was only identified incidentally during a routine pre-surgical consultation. The case underscores the utility of expanding access to genetic diagnostics, which can facilitate the identification of milder or previously overlooked cases within families affected by inherited syndromes.

## 3. Discussion

The symptoms of GGS can involve multiple organ systems, although they predominantly affect structures in the head and neck region [18,19]. Some features of the syndrome can be detected even during prenatal imaging, with orofacial clefts (such as cleft lip or palate) often being the earliest visible abnormalities [8,9,20,21,22,23]. Distinctive craniofacial features may be evident immediately after birth, including frontal bossing and hypertelorism, both considered developmental anomalies [8,9,24,25]. Various dental abnormalities are common, including impacted teeth, hypodontia (tooth agenesis), a high-arched palate, mandibular prognathism, and malocclusion such as an open bite [25]. Externally, dermatological examination may reveal nodular, pearly lesions with rolled edges and central ulceration—features typical of BCC [1,2,3,4,5,6,8,9,11,12,13,14,15]. These signs often prompt referral for dermatological consultation. Radiologically, routine imaging such as OPG may reveal multiple OKCs in the maxilla and mandible, which are considered one of the hallmark features of GGS [1,2,3,5,8,9,11]. Additional findings may include excessive pneumatization of the paranasal sinuses, calcifications of the falx cerebri and sella turcica, and macrocephaly [8,9,25]. These features are diagnostically important and should prompt dental professionals, orthodontists, or maxillofacial surgeons to consider the possibility of a syndromic condition. Early recognition and diagnosis of GGS are crucial as untreated OKCs can expand significantly and, in rare cases, undergo malignant transformation, e.g., squamous cell carcinoma [1,8,9,11,25,26]. A multidisciplinary approach and timely management can substantially reduce the risk of long-term complications.

In our study, we observed two family members with GGS—a mother and her son—who presented markedly different clinical courses despite sharing the same pathogenic variant in the *PTCH1* gene. Over a 22-year follow-up, the son experienced recurring OKCs, requiring more than nine surgical interventions. In contrast, the mother, diagnosed later in life during evaluation for suspected BCC, exhibited predominantly cutaneous BCCs. This intrafamilial phenotypic variability may reflect age-related penetrance, environmental factors, or epigenetic influences affecting gene expression [27]. Such differences highlight the importance of not excluding a diagnosis based solely on the absence of classic symptoms in relatives.

This was a confirmed familial case of Gorlin–Goltz syndrome with autosomal dominant inheritance. The affected mother transmitted the pathogenic variant to her son, which is consistent with the expected 50% inheritance risk. Her daughter remains asymptomatic and is the only first-degree relative who has not undergone genetic testing. While it is possible that she carries the mutation with incomplete penetrance, genetic testing has not been clinically indicated to date. Should she consent to testing in the future, this may offer additional insights and contribute to further publication.

This case underscores the clinical relevance of family history and the importance of considering genetic screening even in the absence of symptoms. The marked differences in average penetrance between mother and son (shown in Table 3) likely reflect not only genetic factors but also phenotypic variability influenced by lesion depth and other modifying conditions. The mother demonstrated higher average penetrance values for *PTCH1* and *PTCH2* compared to her son yet exhibited fewer OKCs, suggesting that lesion depth and anatomic extent may significantly shape clinical presentation. Environmental factors also play a key role: individuals with lighter skin and higher sun exposure are more prone to extensive cutaneous manifestations of GGS and have an increased risk of BCC malignant transformation. This aligns with broader epidemiological observations linking ultraviolet radiation and skin phototype to the severity and progression of basal cell carcinoma in BCNS populations [12,28]. Furthermore, recurrence of OKCs typically diminishes with age. Most recurrences occur within five years of treatment, while late recurrences beyond 40 years of age are rare [29]. In our familial cases, the son’s multiple recurrences over 22 years contrast sharply with the mother’s later onset and more stable oral disease course, underscoring age-dependent penetrance.

Diagnostic limitations should be acknowledged. The mother was screened only for head and neck manifestations; additional phenotypic features such as macrocephaly, skeletal anomalies (e.g., rib bifurcation, vertebral defects), and palmar or plantar pits were not assessed with advanced imaging due to her age and comorbidities (hypertension, type 2 diabetes, asthma, and anemia). Only manual examination ruled out palmar/plantar pits. Thus, her phenotypic profile may be incomplete, potentially underestimating extracutaneous features.

A similar familial pattern was reported by Li et al. [13] in 2024, where a mother and daughter, both of Asian descent, presented with different symptoms, particularly in terms of dermatological manifestations. In their case, the mother was diagnosed first, followed by her daughter, which is in contrast to our report, in which the mother received a definitive diagnosis nearly 20 years later after her son. Other case reports also support the diverse clinical presentation of GGS. One report described a 24-year-old man presenting with facial swelling, mandibular prognathism, nasal flattening, milia, hypertelorism, and ptosis. Imaging revealed calcification of the falx cerebri and rib splaying, and biopsy confirmed the presence of OKCs [30]. Another case involved a 22-year-old man who presented with facial asymmetry, hypertelorism, pectus excavatum, epidermoid cysts, and multiple OKCs [31]. Imaging revealed calcification of the falx cerebri, a bifid rib, and anomalies of the lumbar spine. These cases reinforce the multisystem nature of GGS, with skeletal abnormalities—such as bifid or splayed ribs—reported in 60–75% of cases. Other frequently observed findings include kyphoscoliosis, spina bifida occulta, and shortened metacarpals [31]. Radiographic identification of falx cerebri calcification remains a key diagnostic indicator, along with the high recurrence rate of OKCs [32]. Early diagnosis is essential for monitoring long-term risks, including cutaneous and intracranial malignancies. As GGS is inherited in an autosomal dominant pattern, genetic counselling is critical due to a 50% risk of transmission to offspring [32].

The increasing availability of genetic testing has significantly improved diagnostic timelines. The introduction of NGS between 2004 and 2006 revolutionized clinical genetics by providing greater speed, broader genomic coverage, and improved cost-effectiveness compared to earlier methods such as Sanger sequencing. NGS allows for the simultaneous sequencing of millions of DNA fragments, facilitating rapid detection of both point mutations and small insertions or deletions with high accuracy [33,34]. In our patients, NGS was conducted using Illumina’s sequencing-by-synthesis platform. This method achieves 100% sensitivity for substitutions and approximately 95% for small indels. Each analysis included an internal control, ensuring high specificity and reliability.

Despite the early onset of symptoms in the son, a formal diagnosis was delayed by 15 years. Conversely, the mother—who presented with symptoms 20 years later—received a diagnosis within one month of referral. This striking difference highlights the impact of advancements in genetic testing and increased clinical awareness over time. Earlier diagnosis enables timely treatment, targeted monitoring of at-risk family members, and the avoidance of unnecessary or debilitating interventions.

While numerous English-language publications have described familial cases of GGS [12,13,14,15,16], to our knowledge, this is the first report documenting such an extended follow-up period. In the case of the son, the most burdensome symptom was recurrent OKCs, requiring nine surgical interventions for cyst removal. In contrast, the most pronounced symptoms in the mother were dermatological manifestations of BCC. Differences in symptom severity between the two cases may be attributed to several factors, including age and the potential for disease progression over time. The mother’s primarily dermatological BCC symptoms may reflect a milder or later-onset form of the syndrome. In family members diagnosed with GGS, symptom expression can vary significantly and should not be the sole criterion for excluding a diagnosis through genetic testing.

Patients with GGS require comprehensive, interdisciplinary management involving dental, dermatological, neurological, and ophthalmological specialists. Psychological or psychiatric support may also be necessary, particularly for younger patients who may be affected by the psychosocial impact of chronic illness. Recurrent surgeries, dental extractions, facial changes, and visible skin lesions may contribute to diminished self-esteem, social anxiety, or depression.

In our male patient, ongoing treatment for both dental and dermatological manifestations of GGS over a 22-year period contributed to the development of depressive symptoms. He is currently receiving care from a psychiatrist and psychologist, highlighting the importance of holistic management in syndromic conditions with long-term health implications. In contrast, his mother did not exhibit signs of clinical depression but demonstrated age-appropriate cognitive features such as slowed speech and mild memory deficits. These symptoms were not considered pathological and may have been partially influenced by comorbidities, including type 2 diabetes mellitus, which could affect cerebral perfusion.

There are established management algorithms based on genetic testing that enable diagnosis as early as the prenatal stage [35]. If a familial mutation is known, both prenatal testing and preimplantation genetic diagnosis (PGD) for NBCCS are possible. Most centers acknowledge that the decision to pursue prenatal testing remains an individual choice. It is advisable that parents who opt for such testing have the opportunity to discuss the results in detail with psychologists, especially when testing is considered for the purpose of pregnancy termination rather than for early diagnosis and management planning [35].

Genetic testing in patients presenting with certain clinical features that do not fully meet diagnostic criteria should also be regarded as a tool for early detection. Many patients exhibit clinical manifestations suggestive of BCNS but remain undiagnosed until adulthood. Clinical features considered ‘red flags’ include keratocystic odontogenic tumors (keratocysts), BCCs in individuals under 20 years of age, palmar or plantar pits, lamellar calcification of the falx cerebri, and desmoplastic medulloblastoma in combination with any other major or minor diagnostic criterion [35].

Genetic testing may also be performed as predictive testing in individuals with an affected family member who are at risk but do not fulfill the clinical diagnostic criteria. Given the need for surveillance for potential complications associated with NBCCS—particularly medulloblastoma—during childhood, it is recommended that the genetic status of at-risk individuals be clarified early in life. Parents frequently wish to ascertain the genetic status of their children before initiating screening protocols to avoid unnecessary procedures in children who have not inherited the pathogenic variant [35].

It is of paramount importance to educate both patients and physicians across multiple specialties, including dentists, pediatricians, internists, and dermatologists. The potential implications of genetic testing—such as socio-economic impacts, the need to ensure long-term care, and the organization of surveillance for individuals with a positive result—should be thoroughly discussed in genetic counseling clinics, ideally with psychological support. Furthermore, considering that treatment often involves mutilating surgical interventions, patients may require psychological support throughout the entire course of management.

## 4. Conclusions

GGS presents with a wide spectrum of clinical manifestations, most commonly involving the head and neck region. Early diagnosis is essential for effective management of patients and the reduction of long-term complications. Given the hereditary nature of the syndrome, which frequently affects multiple family members, genetic testing should be considered for all at-risk relatives, regardless of the presence or absence of clinical symptoms.

In our cases, the disease course differed significantly between the mother and son, despite the fact that they shared the pathogenic variant in the *PTCH1* gene. The son experienced a more aggressive course, requiring multiple surgical interventions due to recurrent OKCs. In contrast, the mother’s clinical presentation was dominated by dermatological manifestations, particularly BCCs. This intrafamilial variability in phenotypic expression underscores the clinical heterogeneity of GGS and highlights the need for an individualized approach to diagnosis, treatment, and monitoring—even among genetically related patients.

The emergence of NGS technology has significantly enhanced the speed, accessibility, and accuracy of genetic diagnostics. Timely genetic testing is particularly important in younger patients, as early identification of GGS allows for the implementation of appropriate preventive and therapeutic measures before serious complications arise.

Optimal care for patients with GGS requires a multidisciplinary approach that integrates dental, dermatological, neurological, ophthalmological, psychological and psychiatric care. Given the chronic and often surgically intensive nature of the condition, regular follow-up is essential. Patients frequently face not only physical challenges but also psychological burdens, including anxiety and depression—especially when treatment involves repeated invasive procedures or visible cosmetic changes.

Although there is currently no curative treatment capable of fully preventing symptom manifestation in genetically predisposed individuals, early diagnosis, access to modern diagnostic tools, and coordinated care can significantly improve outcomes and quality of life in patients with GGS. Ongoing surveillance, patient education, and support from a multidisciplinary medical team remain the cornerstone of effective management for this complex syndrome.

## Figures and Tables

**Table 1:** Genetic test results and variant characteristics identified in Case 1.

Patient	Gene	Nucleotide Variant	Genomic Location	Amino Acid Change	Zygosity	Mutation Frequency in EU	Variant Classification
Son	*PTCH1*	NM_000264: c.1067 + 1G>A	Intron 7 of 23	ND	Heterozygous	ND	Pathogenic variant

Abbreviations: EU, European Union; ND, not determined.

**Table 2:** Genetic test results and variant characteristics identified in Case 2.

Patient	Gene	Nucleotide Variant	Genomic Location	Amino Acid Change	Zygosity	Mutation Frequency in EU	Variant Classification
Mother	*PTCH1*	NM_000264: c.1067 + 1G>A	Intron 7 of 23	ND	Heterozygous	ND	Pathogenic variant

Abbreviations: EU, European Union; ND, not determined.

**Table 3:** Comparison of average read depth (penetrance) and quality thresholds for *PTCH1*, *PTCH2*, and *SUFU* genes in a described familial case of GGS.

	Son		Mother	
Gene	Average Penetrance	Quality Threshold	Average Penetrance	Quality Threshold
***PTCH1*** OMIM #601309	59.8	100%	107. 0	97.1%
***PTCH2*** OMIM #603673	44.2	98.5%	114.5	100%
***SUFU*** OMIM #607035	48.8	100%	91.5	97%

Abbreviation: OMIM, Online Mendelian Inheritance in Man (catalog of human genes and genetic disorders and traits).

## Data Availability

The data are available on reasonable request from the corresponding author.

## Abbreviations

BCNS	Basal Cell Nevus Syndrome
NBCCS	Nevoid Basal Cell Carcinoma Syndrome
CT	Computed Tomography
MRI	Magnetic Resonance Imaging
OPG	Orthopantomogram
GGS	Gorlin–Goltz Syndrome
PTCH	Patched
SUFU	Suppressor of Fused
BCC	Basal Cell Carcinoma
OKC	Odontogenic Keratocyst
CNS	Central Nervous System
NGS	Next-Generation Sequencing
